# Melatonin, aging, and COVID-19: Could melatonin be beneficial for COVID-19 treatment in the elderly?

**DOI:** 10.3906/sag-2005-356

**Published:** 2020-10-22

**Authors:** Güler ÖZTÜRK, Kazime Gonca AKBULUT, Şevin GÜNEY

**Affiliations:** 1 Department of Physiology, Faculty of Medicine, İstanbul Medeniyet University, İstanbul Turkey; 2 Department of Physiology, Faculty of Medicine, Gazi University, Ankara Turkey

**Keywords:** Aging, apoptosis, COVID-19, immune system, melatonin, oxidative stress

## Abstract

The aim of this review is to summarize current studies on the relationship between melatonin and aging. Nowadays, age-related diseases come into prominence, and identifying age-related changes and developing proper therapeutic approaches are counted as some of the major issues regarding community health. Melatonin is the main hormone of the pineal gland. Melatonin is known to influence many biological processes in the body, including circadian rhythms, the immune system, and neuroendocrine and cardiovascular functions. Melatonin rhythms also reflect the biological process of aging. Aging is an extremely complex and multifactorial process. Melatonin levels decline considerably with aging and its decline is associated with several age-related diseases. Aging is closely associated with oxidative damage and mitochondrial dysfunction. Free radical reactions initiated by the mitochondria constitute the inherent aging process. Melatonin plays a pivotal role in preventing age-related oxidative stress. Coronavirus disease 2019 (COVID-19) fatality rates increase with chronic diseases and age, where melatonin levels decrease. For this reason, melatonin supplementation in elderly could be beneficial in COVID-19 treatment. Therefore, studies on the usage of melatonin in COVID-19 treatment are needed.

## 1. Introduction

During the aging process, the cell's ability to cope with external and internal stress and its functional capacity is diminished by time. Increased morbidity and mortality rates for several diseases accompany this process. Since aging is an extremely complex and multifactorial process [1], it is impossible to describe it using a single mechanism. Many theories have been proposed to explain the process of aging. However, none of them appear to be entirely convincing. Modern biologic theories of aging in humans can be considered under two main categories: programmed and damage or error theories [2]. The programmed theory has three subcategories: programmed longevity, endocrine theory, and immunological theory. The damage or error theory has several subcategories such as wear and tear theory, rate of living theory, cross-linking theory, free radicals theory, and somatic DNA damage theory [2].

Melatonin [N- acetyl-5-methoxytryptamine] is associated with almost all aging theories and it is primarily released from the pineal gland with a circadian rhythm. Therefore, the pineal gland is prominent in aging theories. In addition retina, lachrymal glands, Harderian gland, gastrointestinal tract, thrombocytes, and bone marrow are other sources of melatonin release [3]. Thus, melatonin is considered to be a wide-spread physiological mediator [4]. Melatonin is also a potent free radical scavenger and affects the immune system [5,6].

## 2. Melatonin, aging, and oxidative stress

Melatonin synthesis decreases during the aging process; a decrease seen in both pineal and plasma levels of melatonin. In rodents, the rate of aging process and onset of age-related diseases can be delayed by exogenous melatonin treatment [5]. The lifespan enhancing effect of melatonin is linked with its antiproliferative feature. Melatonin effects both normal and tumoral proliferation of gastrointestinal cells [7,8].

In terms of the biology of aging, attenuated basal metabolic rate and physiologic performance in elderly mammalians relies largely on mitochondria. Mitochondria are the main source of adenosine triphosphate (ATP) and also play a prominent role in several cellular processes, such as beta oxidation of fatty acids, synthesis of phospholipids, calcium signaling, production of reactive oxygen species (ROS), and apoptosis [9].

Free radical theory of aging explains the aging process based on the functional dysfunction of mitochondria. As is already known, mitochondria are the main site for oxygen consumption, and ROS production; thus, they are also a main target for oxy-radical dependent cellular damage. Attenuated mitochondrial respiration affects lifespan by changing intracellular ROS balance [10,11]. There are some studies which have shown that electron transfer was reduced in mitochondria isolated from elderly animals [12]. Moreover, it has been shown that there is a positive correlation between reduced fluidity of cellular membranes, including mitochondrial membranes, and increased lipid peroxidation during aging [13]. An age-dependent decrease in the activity of manganese superoxide dismutase (Mn-SOD), and increase in the levels of thiobarbituric acid reactive products (TBARS), and carbonyl compounds have been found in brain tissue [14]. Higher oxidation levels indirectly change cellular redox potential, and decreases glutathione (GSH), ATP levels, and reduced NADH/NADPH balance. Due to these alterations, permeability of mitochondrial transition pores (MPTP) increases, and apoptosis is stimulated. It has been reported that protein carbonyl products, TBARS, hydroperoxide, and 8-hydroxydeoxyguanosine levels in brain mitochondria were increased with aging [15].

Due to its lipophilic and hydrophilic properties, melatonin can easily diffuse into all cellular and intracellular compartments, and can pass through the blood-brain barrier. Moreover, no specific binding site or receptor is required for its free radical scavenger effect [16,17]. The pyrrole ring structure of melatonin provides higher capacity to entrap O2•- and •OH radicals. It has been shown that melatonin is five times more effective than endogenous free radical scavenger glutathione, and fifteen times more effective than mannitol, which is an exogenous free radical scavenger. This evidence actually emphasizes that melatonin is a much more powerful antioxidant against •OH radical, compared with the aforementioned antioxidants. The antioxidant effect of melatonin occurs by trapping the O2•- radical via the indolyl cation radical, which is formed by the reaction of melatonin and the •OH radical [5]. Melatonin is found intensively in subcellular compartments, such as the nucleus and mitochondria [18–20]. Mitochondria and mitochondrial dysfunction play a role in the cellular self-destruction processes, including apoptosis, autophagy, and necrosis [21]. The mitochondrial DNA (mtDNA) is more vulnerable than nuclear DNA as it is not enclosed within basic histones. The mtDNA damage in aging humans is 10-fold higher compared with nuclear DNA [21].

Although mitochondria are the main source of ROS formation, mitochondria are also the primary targets of ROS attacks [22]. 

Acuña-Castroviejo et al. reported that age-dependent mitochondrial oxidative stress was prevented by melatonin treatment [23]. Melatonin reduces free radical formation, and protects the cell membrane from free radical attacks. It also plays a pivotal role for mitochondrial homeostasis by optimizing the transfer of electrons through the electron transport chain in the inner mitochondrial membrane [6,19,24,25]. Rodríguez et al. reported that melatonin treatment restored mitochondrial ATP production in heart cells. They also showed that long term melatonin treatment prevents mitochondrial function deficiency and oxidative stress without any side effects caused by the melatonin [26].

It is already known that oxidative stress and the ischemia-reperfusion process take part in the pathogenesis of several diseases, like Alzheimer’s disease, Parkinson’s disease, and Diabetes mellitus [12]. Likewise there is a linear relationship between oxidative stress and aging; aging is a major risk factor for a variety of neurodegenerative diseases [27]. Increased free radical levels in tissues during aging processes have been reported by several studies [28–30].

Gastrointestinal tract derived melatonin is estimated to be approximately 400 times higher than the amount produced by the pineal gland [31]. Studies in mice and rats have shown that melatonin and its specific binding sites are found especially in the colon mucosa [32]. As a broad spectrum antioxidant, and with its free radical scavenging feature, melatonin ameliorates mucosal defense against various irritants and improves gastrointestinal system (GIS) lesions such as esophagitis, gastritis, peptic ulcer, and stomatitis [33]. Callagan et al. showed that hyperplasia developed in the small intestine and colon crypts after pinealectomy [7]. Melatonin has been shown to improve immune functions of the intestine, reduce peristalsis, and regulate fecal water content [31].

Melatonin is known to suppress tumor development. Deterioration of melatonin release has been closely associated with an increased incidence of colorectal cancer. In addition to colorectal cancer, melatonin is also related to the tumor onset, prognosis, and prevention of various cancers of the gastrointestinal tract, such as cholangiocarcinoma, hepatocarcinoma and pancreatic carcinoma. Melatonin exerts its antitumoral effect by decreasing cellular proliferation, autophagy, metastasis, and angiogenesis while activating apoptosis and colon cancer immunity [34].

As aforementioned, melatonin is a rather powerful and effective endogenous radical scavenger [35], and attenuates increased oxidative stress caused by several toxic substances like safrole and paraquat in tissues [36,37].

Besides this, exogenous administration of melatonin also has beneficial effects in terms of oxidative stress. Akbulut et al. previously showed that melatonin treatment prevents age-related oxidative stress, while it increases antioxidant defense in the cerebral cortex and hippocampus [29]. Güney et al. showed that melatonin treatment attenuates H2O2 production and age-related increased lipid hydroperoxide formation in the liver and heart [30].

Nitric oxide (NO), synthesized by nitric oxide synthase (NOS), behaves as either a prooxidant or neuroprotective agent. [38,39]. Since the breakdown of nitric oxide occurs in a very short time, its demolition products nitrate and nitrite are used for measuring the nitric oxide levels [40]. NOx refers to nitric oxide and is obtained by subtracting nitrite from nitrate. Depending on its local concentration or existing cell type, NO can stimulate apoptosis, as well as maintaining cellular survival [41]. NOS activity has been shown in several brain areas, such as the cerebral cortex, cerebellum, hippocampus, and hypothalamus [42]. 

The aging process occurs along with inflammation, oxidative stress, and increased expression of inducible NOS (iNOS) in several tissues [43,44]. The relationship between aging and NO is still contradictory, and needs to be clarified [45] *.* Akbulut et al. showed that NOx levels in temporal cortex increased parallel with age. In the same study they also showed that short term melatonin treatment led to increased NO levels in the cortex; however, it was not effective for the hippocampus [46]. 

Organisms have powerful defense mechanisms against oxidative stress including antioxidant enzymes. Efficacy of antioxidant enzymes, however, diminishes with aging [28,47]. Glutathione is the most important nonprotein thiol compound which balances ROS in brain tissue. GSH redox cycle is essential for the scavenging of cellular free radicals. It maintains homeostasis via de novo glutathione synthesis and redox cycle. It has been reported that age-dependent increased oxidative stress diminished glutathione pools of brain mitochondria by half, and increased oxidized GSH levels [48]. Zhu et al. found that GSH levels were decreased; however, oxidized glutathione (GSSG) and GSSG/GSH levels were increased in several sites in the brain, such as the cortex, hippocampus, striatum, and cerebellum [49]. In several brain areas of rats at different ages, Sandhu et al. reported a decrease in the levels of SOD, GSH, glutathione reductase (GRd), and glutathione peroxidase (GPx) [50]. Even though mitochondrial antioxidant defense mechanisms depend on GSH, mitochondria itself cannot synthesize GSH [51–53]. GSH is taken into mitochondria from cytosol via a multicomponent transporter system [53,54]. Melatonin increases GPx, GRd, SOD, and catalase (CAT) gene expressions and enzyme activities in both pharmacological and physiological doses [55]. During the aging process, mitochondrial GPx levels increase in the brain, while melatonin treatment diminishes mitochondrial GPx levels in both young and elderly rats [56]. Melatonin treatment also enhances mitochondrial SOD levels in elderly rats, and prevents the reduction of SOD/GPx and GR/GPX ratios [56]. Indeed melatonin treatment has been found to increase renal SOD activity and GSH levels and decrease NOx levels in renal ischemia/reperfusion injury in rats [57].

## 3. Melatonin and apoptosis

Oxidative stress is known to stimulate apoptosis, and depending on the tissue type, apoptosis increases by age [58]. Apoptosis is a complicated process involving a cascade mechanism that employs many proteins [59]. The key enzymes in this process are the caspases. Caspases are a family of cysteine proteases widely known as the principal mediators of the apoptotic cell death response [59]. Exogenous melatonin administration has been shown to attenuate age-related increased caspase 3 activity in frontal and temporal cortices, and gastric mucosa of elderly rats [46,60]. Melatonin is known to be effective on antiapoptotic Bcl­2 and apoptotic Bax proteins [61]. An excessive amount of ROS can be produced during aging, or formed by increased metabolic rate or environmental stress. In the presence of excessive ROS, melatonin stimulates ERK1/2 signaling pathway by receptor-dependent or -independent mechanisms, and increases expressions of antioxidants and detoxification genes, like glutathione and its enzymes via stimulation of transcription factors such as Nf­-, AP­-1, and Nrf2 [62]. As a result levels and activity of antioxidant enzymes such as GSH­-Px, GSH-Rd, CAT, and GSH/GSSG ratio were enhanced as well as redox-sensitive Bcl­2 or Bcl­2/Bax ratio. Several studies reported that melatonin, decreased ROS production via controlling the mitochondrial damage and signaling functions of mitochondria, and augmented the activity of antiapoptotic Bcl­2 [63–65]. 

P53 protein is an important transcription factor, related to the maintenance of genomic integrity by controlling the cell cycle progress and cellular survival [66]. It either ceases the progression of the cell cycle or stimulates apoptosis circumstantial with the conditions which cause genomic stress (DNA damage, hypoxia, etc.) [67]. DNA double strand breaks, base modifications, and point mutations may occur because of ROS attacks [68]. In the case of DNA damage, P53 gene expression is increased. Melatonin has different effects on apoptotic processes depending on the cell type. It increases p53 activity in tumor cells while protects other cells such as immune cells or neurons [59,69]. There are some studies about the antiproliferative effect of melatonin on tumor cells. For example Mediavilla et al. showed that melatonin displays an antiproliferative effect on MCF-7 human breast cancer cells by retaining cell cycle and increasing p53 activity [69]. Apoptotic cell death in LNCaP androgen sensitive prostate cancer cells depend on p53. Melatonin increases apoptosis and stimulates p53 and p21 in these cells in a dose dependent manner [70]. However, in some other studies on the effect of melatonin on apoptosis in gastric mucosa during aging, the exogenous melatonin administration on p53 levels was not significant [60].

In an experimental diabetes model, an increase in apoptotic cell number is observed, and downregulation of Bcl-2, attenuation of activities of p53, and caspases 3,8, and 9 have been reported. Melatonin administration throughout 21 days reduced apoptotic cell number and activities of p53, and caspases 3,8,and 9, while it enhanced Bcl­2 levels [71]. Sinanoglu et al. also showed that melatonin pretreatment against renal ischemia/reperfusion injury in rats prevented caspase­3-dependent cell apoptosis. The antioxidant effect of melatonin may partly depend on its antiapoptotic effect [72]

## 4. Melatonin and inflammation

The anti-inflamatory feature of melatonin is explained with several molecular mechanisms, and is related to the modulation of several transcription factors like NF­κB, hypoxia inducible factor, nuclear factor erythroid 2-­related factor 2, and with modulation of iNOS expression. NO is the key molecule for the inflammation. Its reaction with superoxide leads to peroxynitrite formation, which triggers nitration of tyrosine residues in cellular proteins, and hence increases cytotoxicity [73]. As it is shown in several studies, melatonin inhibits the expressions of iNOS and cyclooxygenase. It also reduces the levels of several mediators like leucotriens, cytokines, chemokins, adhesion molecules and reduces production of excessive amount of NO [74–78]. NOS subtypes have different effects in neuropathologic conditions, nNOS and iNOS act together in neurogenerative processes, while eNOS diminishes neuronal injury. Melatonin reduces levels of iNOS and eNOS; however, it increases eNOS levels in a variety of clinical manifestations such as brain ischemia, spinal cord injury, and diabetic neuropathy [79–82]

## 5. Melatonin and the immune system

Both cellular and humoral immune responses decrease with age. As a result of decreased immune system activity people can face increased risk factors, such as cancer, infections, and autoimmune diseases. Due to its anti-inflammatory feature and effects on the immune system, melatonin is one of the most prominent candidates used to explain aging physiopathology [28,83]. Melatonin has been shown to restore age-related decline in immune responses [84]. Notably, the main target of melatonin is the thymus which is the central organ of the immune system [85]. The effects of melatonin on thymus endocrine activity and immune functions are partly dependent on its relation to zinc, which is an essential component of more than 200 enzymes [84,86,87]. Zinc contributes to the age related changes in the immune system, and the zinc pool is altered with aging. Melatonin is reported to regulate zinc turnover. Öztürk et al. showed that liver zinc levels were reduced by age and melatonin recovered age-related decrement in zinc levels of the thymus tissue in elderly rats [87]. Moreover, in another study, melatonin treatment has been shown to increase zinc level in the small intestines of young rats during night time, while it increased zinc levels in salivary glands during day and night time via the zinc absorption from small intestines [88].

With age, functional activity of natural killer (NK) cells, lymphokine secretion, in particular IL­-2 synthesis and IL-­2 receptor expression, diminish together with T cell response [89,90]. Aging decreases humoral immunity; however, decrement in cellular immunity is more specific. Melatonin treatment enhances Ig and IL­-2 responses. Akbulut et al. reported that melatonin administration to elderly rats increased IgG and IgM levels significantly [91]. Inhibition of melatonin synthesis in mice, attenuates primary antibody response, and decreases cell numbers in the spleen and thymus [85].

## 6. Melatonin and coronavirus disease 2019

In December 2019, the third pathogenic human coronavirus (HCoV) was identified as the cause of coronavirus disease 2019 in Wuhan, China. Coronaviruses (CoVs) are RNA viruses infecting both human and animals. They typically affect the respiratory tract of mammals, including humans, and lead to mild to severe respiratory tract infections [92]. The world is in the grip of the coronavirus disease 2019 (COVID­-19) pandemic and COVID-­19 continues to spread to all countries. No specific treatment has yet been found for COVID­-19. Vaccination and drug studies are in progress.

Viral infections are often associated with immune-inflammatory injury, in which the level of oxidative stress increases significantly and has negative effects on the function of multiple organs [92]. Melatonin plays a key role in several biological processes, and offers an alternative point of view in the management of viral infections. Even though melatonin cannot eradicate or even curb the viral replication or transcription, due to its anti-inflammatory and antioxidant effects, melatonin has been suggested as a candidate drug to relieve patients’ clinical symptoms in antiviral treatment [92,93]. In addition, it has been suggested that melatonin treatment may prolong patients' survival time, which may provide a chance for recovery of the immune system and eventually eradication of the virus. Melatonin indirectly targets several HCoV cellular targets, including ACE2, BCL2L1, JUN, and IKBKB [92]. Therefore, its usage in COVID­-19 treatment might be beneficial. COVID-19 fatality rates increase with chronic diseases and age, where melatonin levels are low.

Melatonin supplementation can reduce the risk of influenza and COVID­-19 incidence. Several studies have proved the positive effects of melatonin in attenuating acute respiratory stress induced by virus, bacteria, and radiation [93]. It has been suggested that melatonin may have supportive adjuvant utility in treating COVID­-19 induced pneumonia, acute lung injury (ALI), and acute respiratory distress syndrome (ARDS). In fact melatonin has been found to reduce the proinflammatory cytokines that trigger inflammation and pneumonia in lungs [93].

A cytokine storm also prevails in patients with COVID-­19. When compared to that of SARS patients, interleukin 1β (IL-­1β), interferon γ (IFN­γ), interferon-inducible (IP-10), and monocyte chemoattractant protein 1 (MCP­1), IL-­4 and IL-­10 levels increased significantly in the blood of patients with COVID-­19. A potential repressed immune function is seen in COVID-­19 patients with the hypoalbuminemia, lymphopenia, and neutropenia, and also there is a decreased percentage of CD8+ T cell. A T-helper-2 (Th2) product IL-10 is an antiviral and coronaviruses lead to a significant decrease in this agent. As a result, inflammation is a major feature in COVID­-19 patients. Activated cytokine storm, depressed immune system, and excessive inflammation may contribute to the pathogenesis of COVID-­19. Thus, overproduction of these cytokines and chemokines contributes to the prognosis of the disease [93]. 

Experimental SARS­-CoV models support this cytokine storm hypothesis related to coronaviruses. One of those experimental models revealed that the severity of ALI was accompanied by an elevated expression of inflammation-related genes rather than increased viral titers. Therefore, Zhang et al. suggested that attenuation of the cytokine storm may provide better results in treatment. [93]. 

Because of its anti-inflammatory, antioxidative, and immune enhancing features, melatonin may have indirect antiviral actions. [94]. The decrease in pineal and mitochondrial melatonin contributes to an increase in the replication and severity of many viral infections [95]. Indeed, melatonin treatment has been shown to alleviate the symptoms of infection and decrease virus load in mice infected with encephalitis viruses. 

SARS-­CoV­2 is believed to cause severe lung pathology by triggering pyroptosis, a highly inflammatory form of programmed cell death. [96]. Pyroptosis in immune system cells can lead to symptoms like lymphopenia that blocks an effective immune response to the virus. It has been proposed that programmed cell death caused by coronaviruses can be inhibited by melatonin. Thus, melatonin intake can improve the protective mechanisms of the body against infections. [96]. 

Ventilation has been reported to increase pulmonary inflammation in acute lung injury and increased oxidative stress in alveoli. As mentioned above, because of the highly effective role of melatonin against oxidative stress, it is reported that melatonin can help to resolve the contradiction between the urgent clinical necessity to deliver mechanical ventilation to a patient and the threat that ventilation may have [96]. As a result, the antiinflammation, antioxidation, and immune enhancing effects of melatonin might be useful in reducing the risk of COVID­-19. 

Moreover, melatonin has been shown to be a vasodilator in the pulmonary arteries, and this effect of melatonin has been reported to be very fast [96]. Due to its vasodilating effect on pulmonary vascular vessels, the role of melatonin in the treatment of COVID-­19 should be investigated and supported by scientific data [97].

## 7. Conclusion

The pineal gland is considered to be the body’s "biological clock", and melatonin is the chief secretory product of this gland. Melatonin plays an important role in many processes in the body, especially in healthy aging and prevention of free-radical-related diseases. Melatonin, as an endogenous synchronizer, affects the regulation of the other hormonal rhythms. The decrease in melatonin production and altered melatonin rhythms with aging can lead to an inhibited immune system. These changes can cause an increase in risk of disease in elderly. Melatonin could reduce the risk of COVID­-19 in the elderly with a weak immune system. The use of melatonin as an antiviral immunostimulant should be supported by studies.
